# Effect of diabetes mellitus and glycemic control on the prognosis of non-muscle invasive bladder cancer: a retrospective study

**DOI:** 10.1186/s12894-020-00684-5

**Published:** 2020-08-05

**Authors:** Wei-Lun Huang, Kuo-How Huang, Chao-Yuan Huang, Yeong-Shiau Pu, Hong-Chiang Chang, Po-Ming Chow

**Affiliations:** Department of Urology, National Taiwan University Hospital, National Taiwan University, College of Medicine, No.7, Chung-Shan South Road, Zhongzheng District Taipei, 100 Taiwan

**Keywords:** Bladder cancer, Urothelial carcinoma, Diabetes mellitus, Glycemic control, Prognosis, Recurrence

## Abstract

**Background:**

Hyperglycemia is associated with series of process leading to oncogenesis. Evidence has shown that diabetes mellitus (DM) seems to be associated with poor prognosis in patients with bladder cancer. However, evidence on the effect of glycemic control on the outcomes of bladder cancer is still limited. In the current study, we aimed to investigate the effect of DM and glycemic control on the prognosis of bladder cancer.

**Methods:**

We conducted a retrospective chart review of a prospective database from January 2012 to December 2017. Patients with newly diagnosed non-muscle invasive bladder cancer (NMIBC) were included. They were classified into the DM and non-DM groups. Prognosis including recurrence rate, progression rate, recurrence-free survival (RFS), and progression-free survival was compared between the two groups. Subgroup analysis of the DM subgroup, in which patients were classified by HbA1C level, was conducted to investigate the effect of glycemic control.

**Results:**

A total of 287 patients were included in our study, with 61 patients in the DM group and 226 patients in the non-DM group. No statistically significant difference was found in the prognosis between the DM and non-DM groups. Subgroup analysis revealed higher recurrence rate (*P* = 0.037) and worse RFS (log-rank *P* = 0.019) in patients with HbA1C ≥ 7.

**Conclusions:**

DM is not a risk factor for recurrence and progression in patients with NMIBC. However, poor glycemic control is associated with poor prognosis in patients with both DM and NMIBC. Further prospective studies are needed to confirm current results.

## Background

Bladder cancer is the tenth most common cancer worldwide, accounting for 3% of all cancers. Approximately 75% of the newly diagnosed bladder cancers are non-muscle invasive bladder cancer (NMIBC; stage Ta, carcinoma in situ (CIS), and T1). The recurrence rates of NMIBC are 61.1% in 2 years, 69.5% in 5 years, and 74.3% in 10 years, and the progression rates are 5–40% for Ta diseases and up to 30–50% for T1 diseases [1, 2].

Diabetes mellitus (DM) is another noteworthy health problem worldwide, with a global prevalence of 8.8% in 2017 [3]. Complications of DM, such as cardiovascular disease, nephropathy, and neuropathy, have been widely discussed. Moreover, cancer is another associating factor for morbidity and mortality. Evidence has shown that DM may be associated with higher incidence and poor prognosis of bladder cancer [4–9]. Furthermore, poor glycemic control results in increase of oxidative stress, upregulation of series of cell molecules, and inflammation process, which are thought to have negative effect on cancer prognosis. However, evidence on the effect of glycemic control on the outcomes of bladder cancer is limited. Besides, most studies use single HbA1C data for evaluation, which may not be representative of long-term glycemic control during the follow-up period. Therefore, in the current study, we aimed to investigate the effect of DM and glycemic control on the outcomes of NMIBC by using mean HbA1C data.

## Methods

### Ethic approval

The present study followed all standards for ethics with regard to experimentation and research. The institutional review board of National Taiwan University Hospital approved our study (approval number: 201901119RINA) and waived the informed consent requirement due to the retrospective design of the study.

### Definition

Patients who had DM or who were diagnosed with DM during the follow-up period were classified into the DM group, and those who had no evidence of DM were classified into the non-DM group. We obtained HbA1C data from patients who received treatments of DM in our hospital and calculated mean HbA1C levels by averaging the sum of HbA1C data from the time of diagnosis of bladder cancer to the time of the first recurrence. We defined patients who had mean HbA1C ≤ 7 as proper glycemic control and those who had mean HbA1C ≥ 7 as poor glycemic control. Diagnosis of bladder urothelial carcinoma was based on pathologic evidence. Cancer staging was based on the 7th edition of the American Joint Committee on Cancer. We determined T stage according to pathologic reports of surgical specimens. Lymph node and distant metastasis were detected using computed tomography or magnetic resonance imaging. Recurrence was defined based on pathologic evidence of surgical specimens that were obtained during follow-up cystoscopy and subsequently confirmed as urothelial carcinoma by pathologists. Progression was defined according to the International Bladder Cancer Group definition as [1] increase in T stage from CIS or Ta to T1 (lamina propria invasion), [2] development of T2 or greater or lymph node (N+) disease or distant metastasis (M1), or [3] an increase in grade from low to high. Recurrence-free survival (RFS) was defined as the period from the date of the initial transurethral resection of bladder tumor (TURBT) to the date of the operation in which the first cancer recurrence was found. Progression-free survival (PFS) was defined as the period from the date of the initial TURBT to the date of the operation or image study in which the first cancer progression was found.

### Patient selection

We enrolled patients who received TURBT. The patients were subsequently diagnosed with NMIBC in our hospital from January 2012 to December 2017. Patients who had newly diagnosed NMIBC with a follow-up period of more than 2 years were included. Patients who had following condition were excluded: [1] lack of the first operative or pathologic report, [2] upper tract urothelial carcinoma (UTUC), which was diagnosed before, concurrently, or after the initial diagnosis of the bladder cancer, [3] muscle invasive bladder cancer, [4] advanced operation for the bladder cancer, e.g., cystectomy, [5] any metastatic cancer, or [6] bladder cancer other than urothelial carcinoma.

### Treatment and follow-up protocols

In our center, resident doctors must receive a 5-year training program for urological specialist. In the first and the second years, junior resident doctors should be trained as assistants and taught by seniors. They should learn the procedure skills by watching and simulation. Well-trained senior resident doctors could conduct TURBT; either senior or junior resident doctors could perform cystoscopy. For pursuing quality and safety of surgeries, all procedures must be supervised by attending doctors in charge. Repeated TURBT was regularly conducted for all patients within 3 months after the first TURBT. For all patients diagnosed with CIS, we conducted mapping biopsies during the follow-up period. Intravesical therapy would be given if no contraindication existed, and the first dose would be given within 24 h after the operation. The follow-up protocol in our center strictly met the current Urological Association (AUA) and European Association of Urology (EAU) guidelines for bladder cancer.

### Study design

We retrospectively analyzed data from a prospective database. Patient profiles and disease characteristics, including age at the time of diagnosis, sex, body mass index (BMI), history of smoking, comorbidities, cancer stage, histology grade of urothelial carcinoma, tumor number, tumor size, intravesical therapy, date of diagnosis, date of recurrence, and date of progression were collected. We divided the patients into the non-DM and DM groups. Subgroup analysis for the DM group, which was further divided into the proper glycemic control and poor glycemic control groups, was performed to investigate prognostic factors in the DM group. Outcomes were recurrence, progression, RFS, and PRS.

### Statistical analysis

Data were analyzed using SPSS version 22 (SPSS Inc., Chicago, IL, USA). Categorical variables were analyzed using Chi-squared test; RFS and PFS were analyzed using Kaplan-Meier analysis. Factors including age, sex, history of smoking, BMI, hypertension, serum creatinine level, DM, glycemic control, metformin use, thiazolidinedione (TZD) use, clinical T1 stage, grade of urothelial carcinoma, concurrent CIS, tumor number, tumor size, and intravesical therapy were analyzed using univariate Cox proportional hazards regression to determine risk factors for recurrence and progression. DM and confounders with *P* <  0.2 in the univariate Cox proportional hazards regression were candidates for multivariate Cox proportional hazards regression to determine independent risk factors for recurrence. In all cases, two-tailed *P* <  0.05 was considered statistically significant.

## Results

### Patient selection and baseline characteristics

We screened 845 patients who had a diagnosis of bladder cancer. As a result, 287 patients had NMIBC with a follow-up period of more than 2 years. Of all these patients, 61 patients (21.3%) had DM, and 226 patients (78.7%) did not have DM (Supplementary Fig. S1).

The median age at diagnosis of bladder cancer was 67 years. The median follow-up period was 45 months. Recurrence and progression were observed after the initial diagnosis in 109 patients (38.0%) and 18 patients (6.3%), respectively. Characteristics were similar between the DM and non-DM groups, except that the patients in the DM group were older and had higher rates of obesity (*P* < 0.01), hypertension (*P* < 0.01), and renal insufficiency (*P* = 0.04) (Supplementary Table S1).

### Prognosis analysis

**Fig. 1:**
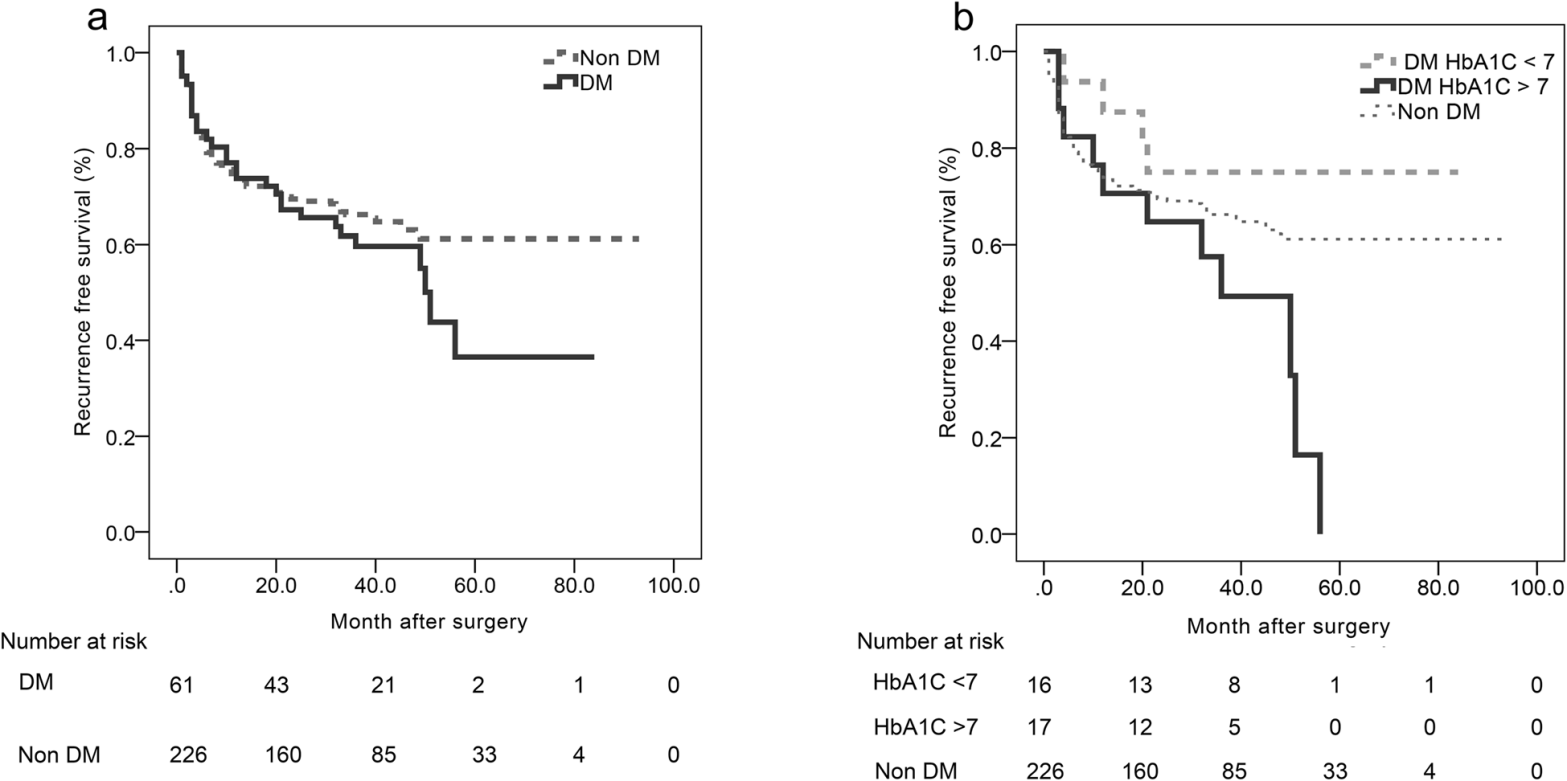
Kaplan-Meier analysis of RFS. **a** RFS in the DM (median 51.00 months, 95% CI 47.77–54.23) and non-DM groups (median not reach), log-rank *P* = 0.221. **b** RFS in the non-DM group (median not reach), proper glycemic control group (median not reach), and poor glycemic control group (median 36.00 months, 95% CI 17.36–54.64), pairwise comparisons: non-DM group vs. poor glycemic control group, log-rank *P* = 0.049; non-DM group vs. proper glycemic control group, log-rank *P* = 0.329; proper glycemic control group vs. poor glycemic control group, log-rank *P* = 0.019

DM was not significantly associated with higher rates of recurrence (odds ratio (OR) = 1.52, 95% confidence interval (CI) 0.86–2.69, *P* = 0.15) and progression (OR = 0.73, 95% CI 0.20–2.60, *P* = 0.62) (Supplementary Table S1). Kaplan-Meier analysis of RFS and PFS revealed no significant difference between the DM and non-DM groups (Fig. 1a and Supplementary Fig. S2a). Univariate Cox proportional hazards regression showed that male sex (hazard ratio (HR) = 1.94, 95% CI 1.14–3.30, *P* = 0.014), T1 stage (HR = 2.14, 95% CI 1.47–3.12, *P* < 0.001), CIS (HR = 1.58, 95% CI 1.03–2.41, *P =* 0.036), high grade (HR = 1.72, 95% CI 1.14–2.58, *P =* 0.010), tumor number ≥ 3 (HR = 2.49, 95% CI 1.58–3.94, *P <* 0.001), and tumor size ≥3 (HR = 1.94, 95% CI 1.12–3.36, *P =* 0.018) were associated with higher recurrence (Table 1). Multivariate Cox proportional hazards regression showed that T1 stage (HR = 2.05, 95% CI 1.06–3.97, *P* = 0.034), tumor number ≥ 3 (HR = 3.46, 95% CI 1.90–6.33, *P <* 0.001), and tumor size (HR = 1.90, 95% CI 1.05–3.42, *P =* 0.033) were independent risk factors for recurrence (Table 1).

**Table 1:** Cox proportional hazards regression for RFS

	Whole cohort (a)										DM subgroup (b)				
	Univariate					Multivariate					Univariate				
	HR	95.0% CI			*P*	HR	95.0% CI			*P*	HR	95.0% CI			*P*
Age	0.86	0.59	–	1.25	0.415						0.94	0.84	–	1.06	0.306
Sex	1.94	1.14	–	3.30	0.014*	1.66	0.71	–	3.88	0.245	4.13	0.98	–	17.44	0.054
Smoking	1.34	0.91	–	1.96	0.136	0.84	0.46	–	1.52	0.558	3.438	0.311	–	38.06	0.314
BMI > 24 kg/m^2^	1.1	0.75	–	1.62	0.610						1.61	0.65	–	3.98	0.301
Hypertension	0.74	0.51	–	1.08	0.113	1.12	0.60	–	2.06	0.726	0.67	0.25	–	1.77	0.418
Cre > 1.5 mg/dL	1.03	0.60	–	1.77	0.921						1.04	0.42	–	2.56	0.936
Dialysis	0.88	0.28	–	2.78	0.831						1.57	0.21	–	11.7	0.658
History of other cancers	0.71	0.36	–	1.41	0.335						0.63	0.15	–	2.69	0.536
cT1	2.14	1.47	–	3.12	< 0.001*	2.05	1.06	–	3.97	0.034*	1.83	0.87	–	3.86	0.112
CIS	1.58	1.03	–	2.41	0.036*	0.69	0.34	–	1.39	0.297	1.34	0.6	–	2.96	0.476
High grade	1.72	1.14	–	2.58	0.010*	0.85	0.43	–	1.68	0.643	1.45	0.61	–	3.42	0.400
Tumor number ≥ 3	2.49	1.58	–	3.94	< 0.001*	3.46	1.90	–	6.33	< 0.001*	0.99	0.41	–	2.41	0.982
Tumor size ≥3 cm	1.94	1.12	–	3.36	0.018*	1.90	1.05	–	3.42	0.033*	1.94	0.64	–	5.83	0.239
Intravesical therapy	0.97	0.36	–	2.64	0.958						21.8	< 0.01	–	> 11.00	0.790
DM	1.3	0.85	–	2.00	0.229	1.11	0.57	–	2.19	0.755					
Urine sugar ≥100											1.86	0.87	–	3.97	0.108
HbA1c ≥7											3.64	1.14	–	11.65	0.029*
Metformin											1.36	0.60	–	3.10	0.460
TZD											0.26	0.04	–	1.90	0.184

a. Whole cohort: Male sex, T1 stage, CIS, high grade, tumor number ≥ 3, and tumor size ≥3 were associated with higher recurrence in univariate analysis. T1 stage, tumor number ≥ 3, and tumor size ≥3 were independent risk factors for recurrence in multivariate analysis

b. DM subgroup: HbA1c ≥7 was associated with higher risk of recurrence

*CI* confidence interval, *CIS* carcinoma in situ, *DM* diabetes mellitus; *HR* hazard ratio, *RFS* recurrence-free survival, *TZD*s thiazolidinedione; * *P* < 0.05

### Subgroup analysis

Patients in the DM group were classified by HbA1C level. Thirty-three patients had medical treatments for DM in our cohort. Among these patients, 16 had proper glycemic control (HbA1C ≤ 7) and 17 had poor glycemic control (HbA1C ≥ 7). The characteristics between two groups were similar, except that renal insufficiency rate was higher in the poor glycemic control group (Supplementary Table S2). The recurrence rate was higher in the poor glycemic control group (OR = 5.50, 95% CI 1.22–24.81, *P =* 0.037) compared with that in the proper glycemic control group. Kaplan-Meier analysis showed that poor glycemic control was also associated with worse RFS (Fig. 1b, log-rank *P* = 0.019); progression rates and PFS were similar between the two groups (Supplementary Fig. S2b). Univariate Cox regression for RFS revealed that only HbA1C ≥ 7 was associated with worse RFS (HR = 3.64, 95% CI 1.14–11.65, *P =* 0.029). Urine glycemic level, metformin use, and TZD use were not associated with RFS (Table 1). We also compared the RFS between the non-DM group, the proper glycemic control group, and the poor glycemic control group by using Kaplan-Meier analysis (Fig. 1b). The RFS was worse in the poor glycemic control group compared with the other two groups. RFS was not significantly different between the non-DM and proper glycemic control groups.

## Discussion

In our study, recurrence and progression rates were not significantly different between the DM and non-DM groups. Metformin and TZD use had no effect on recurrence rate. Nevertheless, in the subgroup analysis, patients who had HbA1C ≥ 7 had significantly higher risks of recurrence and worse RFS. Patients with DM who had proper glycemic control had similar RFS compared with patients without DM.

DM is associated with increasing incidence and poor prognosis of several cancers, including colorectal, breast, endometrial, liver, pancreatic, and bladder cancers [4, 5, 10–13]. Several studies have discussed the effect of DM on bladder cancer. Xu et al. conducted a meta-analysis including 21 cohort studies, which involved 13 million participants, and reported that DM is associated with a higher risk of bladder cancer or cancer mortality (relative risk: 1.23; 95% CI = 1.12–1.35) [14]. Other studies also reported higher recurrence of NMIBC in patients with DM, with rates of 45–60% in patients with DM versus 30–40% in patients without DM [6–8].

Another interesting issue is whether glycemic control is associated with prognosis of bladder cancer. Hwang et al. found that patients who had serum HbA1C ≥ 7 have higher rates of tumor multiplicity and tumor grade, but the recurrence and progression rates are not significantly higher [7]. Ahn et al. reported that poor glycemic control is associated with a higher progression rate and worse PFS [8]. Tai et al. also reported that poor glycemic control is associated with a higher risk of bladder recurrence in patients with UTUC [15]. However, the HbA1C levels for analyses in those studies were single data obtained during the study period rather than averages of all data in the study period.

Metformin has been discovered to suppress tumor by activating AMP-activated protein kinase and liver kinase B1 and downregulating mammalian target of rapamycin and insulin-like growth factor-1 [16]. Tseng reported that cumulative dose and duration of metformin use are associated with decreased incidence of bladder cancer [17]. Studies also found that metformin use appears to be associated with better RFS or cancer-specific survival in patients with bladder cancer [6, 18, 19]. However, the effect of metformin on reducing the incidence and recurrence of bladder cancer were challenged by other studies [8, 20, 21]. On the other hand, pioglitazone is associated with increased risk of bladder cancer [22, 23].

The mechanism of association between bladder cancer and DM remains unclear. Chronic inflammation and hyperinsulinemia induced by hyperglycemia may be the two major factors. Poor glycemic control results in direct cell damage by fluctuating serum glucose level and accumulation of advanced glycation end products (AGEs). The interaction between AGEs and their receptors leads to increased oxidative stress that results in DNA damage, upregulation of series of cell molecules, and inflammation process. Cell molecules including transcription factors (e.g., NF-κB, STAT3, HIF1α) and cytokines (e.g., IL-6, Cox-2, and TNF-α) coordinate together and lead to the amplification of inflammation and creation of a suitable environment for cancer growth [24–26]. Hyperinsulinemia in type 2 DM upregulates insulin-like growth factors (IGF) that act as stimulators of mitogenesis and cellular transformation. On the other hand, insulin-like growth factor-binding proteins (IGFBPs) serve as moderators of IGFs and regulate cell proliferation and apoptosis [27]. Zhao et al. reported that patients with bladder cancer have higher plasma levels of IGF-1 and lower levels of IGFBP-3 than patients without bladder cancer [28]. Studies have also reported that overexpression of IGFs and their binding proteins is associated with poor prognosis in bladder cancer [29, 30].

This study has several limitations. First, the DM group had fewer patients, and only 33 patients had HbA1C data. Due to a small sample size, there is a lack of multivariate analysis bias in the subgroup analysis. Second, demographic distributions in the DM and non-DM groups were not similar in terms of age, obesity, hypertension, and serum creatinine level, which may lead to potential biases.

On the other hand, several strengths of our study are noteworthy. First, for patients who had HbA1C data, we calculated the mean HbA1C levels from the time of diagnosis to the time of end points. To our knowledge, this study is the first to use mean HbA1C levels for evaluation, which is more representative because the follow-up period of cancer may be much longer than the half-life of HbA1C. Second, we included patients with a follow-up period of more than 2 years, in which more than 80% of recurrence occurred. Third, we performed Kaplan-Meier analysis for patients without DM, patients with DM and proper glycemic control, and patients with DM and poor glycemic control. The result shown in Fig. 1b illustrates the relationship between glycemic control and RFS. Finally, in our center, treatment and follow-up protocol for bladder cancer were strictly according to the current AUA or EAU guidelines. For patients with CIS, mapping biopsies were regularly performed. We believe that the detection of recurrence or progression can be early as possible.

## Conclusions

The results of our study suggest that DM is not a risk factor for recurrence and progression in patients with NMIBC. However, poor glycemic control is associated with a higher rate of recurrence and worse RFS in patients with DM. Therefore, proper glycemic control should be one of treatment goals in patients with DM and NMIBC. In addition, due to small sample size and retrospective design, further prospective studies are needed to confirm current results.

## Supplementary information

**Additional file 1: Figure S1.** The flowchart of patient selection. **Figure S2.** Kaplan-Meier analysis of PFS. **Table S1.** Characteristics of the DM and non-DM groups. **Table S2.** Characteristics in the proper glycemic control group and the poor glycemic control group.

## Data Availability

The datasets generated during and/or analysed during the current study are available from the corresponding author on reasonable request.

## Abbreviations

AGE: Advanced glycation end products
AUA: American Urologic Association
BCG: Bacillus Calmette–Guérin
CIS: Carcinoma in situ
DM: Diabetes mellitus
EAU: European Association of Urology
HR: Hazard ratio
IGF: Insulin-like growth factors
IGFBP: Insulin-like growth factor-binding protein
MIBC: Muscle invasive bladder cancer
NMIBC: Non-muscle invasive bladder cancer
OHA: Hypoglycemic agent
OR: Odds ratio
PFS: Progression-free survival
RFS: Recurrence-free survival
TURBT: Transurethral resection of bladder tumor
TZD: Thiazolidinedione
UTUC: Upper urinary tract urothelial carcinoma

## References

[1] Bray F, Ferlay J, Soerjomataram I, Siegel RL, Torre LA, Jemal A (2018). Global cancer statistics 2018: GLOBOCAN estimates of incidence and mortality worldwide for 36 cancers in 185 countries. CA Cancer J Clin.

[2] Donat SM (2003). Evaluation and follow-up strategies for superficial bladder cancer. Urol Clin N Am.

[3] IDF Diabetes Atlas, 8th edn. International Diabetes Federation; Brussels, Belgium: International Diabetes Federation. 2017.

[4] Coughlin SS, Calle EE, Teras LR, Petrelli J, Thun MJ (2004). Diabetes mellitus as a predictor of cancer mortality in a large cohort of US adults. Am J Epidemiol.

[5] Tseng CH, Chong CK, Tseng CP, Chan TT (2009). Age-related risk of mortality from bladder cancer in diabetic patients: a 12-year follow-up of a national cohort in Taiwan. Ann Med.

[6] Rieken M, Xylinas E, Kluth L, Crivelli JJ, Chrystal J, Faison T (2013). Association of diabetes mellitus and metformin use with oncological outcomes of patients with non-muscle-invasive bladder cancer. BJU Int.

[7] Hwang EC, Kim YJ, Hwang IS, Hwang JE, Jung SI, Kwon DD (2011). Impact of diabetes mellitus on recurrence and progression in patients with non-muscle invasive bladder carcinoma: a retrospective cohort study. Int J Urol.

[8] Ahn JH, Jung SI, Yim SU, Kim SW, Hwang EC, Kwon DD (2016). Impact of glycemic control and metformin use on the recurrence and progression of non-muscle invasive bladder Cancer in patients with diabetes mellitus. J Korean Med Sci.

[9] MacKenzie T, Zens MS, Ferrara A, Schned A, Karagas MR (2011). Diabetes and risk of bladder cancer: evidence from a case-control study in New England. Cancer..

[10] Giovannucci E, Harlan DM, Archer MC, Bergenstal RM, Gapstur SM, Habel LA (2010). Diabetes and cancer: a consensus report. Diabetes Care.

[11] Larsson SC, Orsini N, Brismar K, Wolk A (2006). Diabetes mellitus and risk of bladder cancer: a meta-analysis. Diabetologia..

[12] Chen Y, Wu F, Saito E, Lin Y, Song M, Luu HN (2017). Association between type 2 diabetes and risk of cancer mortality: a pooled analysis of over 771,000 individuals in the Asia cohort consortium. Diabetologia..

[13] Rao Kondapally Seshasai S, Kaptoge S, Thompson A, Di Angelantonio E, Gao P, Sarwar N (2011). Diabetes mellitus, fasting glucose, and risk of cause-specific death. N Engl J Med.

[14] Xu Y, Huo R, Chen X, Yu X (2017). Diabetes mellitus and the risk of bladder cancer: a PRISMA-compliant meta-analysis of cohort studies. Medicine (Baltimore).

[15] Tai YS, Chen CH, Huang CY, Tai HC, Wang SM, Pu YS (2015). Diabetes mellitus with poor glycemic control increases bladder cancer recurrence risk in patients with upper urinary tract urothelial carcinoma. Diabetes Metab Res Rev.

[16] Pernicova I, Korbonits M (2014). Metformin--mode of action and clinical implications for diabetes and cancer. Nat Rev Endocrinol.

[17] Tseng CH (2014). Metformin may reduce bladder cancer risk in Taiwanese patients with type 2 diabetes. Acta Diabetol.

[18] Nayan M, Bhindi B, Yu JL, Hermanns T, Mohammed A, Hamilton RJ (2015). The effect of metformin on cancer-specific survival outcomes in diabetic patients undergoing radical cystectomy for urothelial carcinoma of the bladder. Urol Oncol.

[19] Rieken M, Xylinas E, Kluth L, Crivelli JJ, Chrystal J, Faison T (2014). Effect of diabetes mellitus and metformin use on oncologic outcomes of patients treated with radical cystectomy for urothelial carcinoma. Urol Oncol.

[20] Mamtani R, Pfanzelter N, Haynes K, Finkelman BS, Wang X, Keefe SM (2014). Incidence of bladder cancer in patients with type 2 diabetes treated with metformin or sulfonylureas. Diabetes Care.

[21] Goossens ME, Buntinx F, Zeegers MP, Driessen JH, De Bruin ML, De Vries F (2015). Influence of metformin intake on the risk of bladder cancer in type 2 diabetes patients. Br J Clin Pharmacol.

[22] Turner RM, Kwok CS, Chen-Turner C, Maduakor CA, Singh S, Loke YK (2014). Thiazolidinediones and associated risk of bladder cancer: a systematic review and meta-analysis. Br J Clin Pharmacol.

[23] Lewis JD, Habel LA, Quesenberry CP, Strom BL, Peng T, Hedderson MM (2015). Pioglitazone use and risk of bladder Cancer and other common cancers in persons with diabetes. JAMA..

[24] Federico A, Morgillo F, Tuccillo C, Ciardiello F, Loguercio C (2007). Chronic inflammation and oxidative stress in human carcinogenesis. Int J Cancer.

[25] Mantovani A, Allavena P, Sica A, Balkwill F (2008). Cancer-related inflammation. Nature..

[26] Chang SC, Yang WV (2016). Hyperglycemia, tumorigenesis, and chronic inflammation. Crit Rev Oncol Hematol.

[27] Grimberg A, Cohen P (2000). Role of insulin-like growth factors and their binding proteins in growth control and carcinogenesis. J Cell Physiol.

[28] Zhao H, Grossman HB, Spitz MR, Lerner SP, Zhang K, Wu X (2003). Plasma levels of insulin-like growth factor-1 and binding protein-3, and their association with bladder cancer risk. J Urol.

[29] Gonzalez-Roibon N, Kim JJ, Faraj SF, Chaux A, Bezerra SM, Munari E (2014). Insulin-like growth factor-1 receptor overexpression is associated with outcome in invasive urothelial carcinoma of urinary bladder: a retrospective study of patients treated using radical cystectomy. Urology.

[30] Szarvas T, vom Dorp F, Niedworok C, Melchior-Becker A, Fischer JW, Singer BB (2012). High insulin-like growth factor mRNA-binding protein 3 (IMP3) protein expression is associated with poor survival in muscle-invasive bladder cancer. BJU Int.

