# Supplementary material for: Effect of diabetes mellitus and glycemic control on the prognosis of non-muscle invasive bladder cancer: a retrospective study
Source: BMC Urol. 2020 Aug 5;20:117. doi: 10.1186/s12894-020-00684-5 (PMC7409398; doi:10.1186/s12894-020-00684-5)
Supplement: Supplementary file 1 — Additional file 1: Figure S1. The flowchart of patient selection. Figure S2. Kaplan-Meier analysis of PFS. Table S1. Characteristics of the DM and non-DM groups. Table S2. Characteristics in the proper glycemic control group and the poor glycemic control group. [file 12894_2020_684_MOESM1_ESM.pdf]

FIG. S1 The flowchart of patient selection

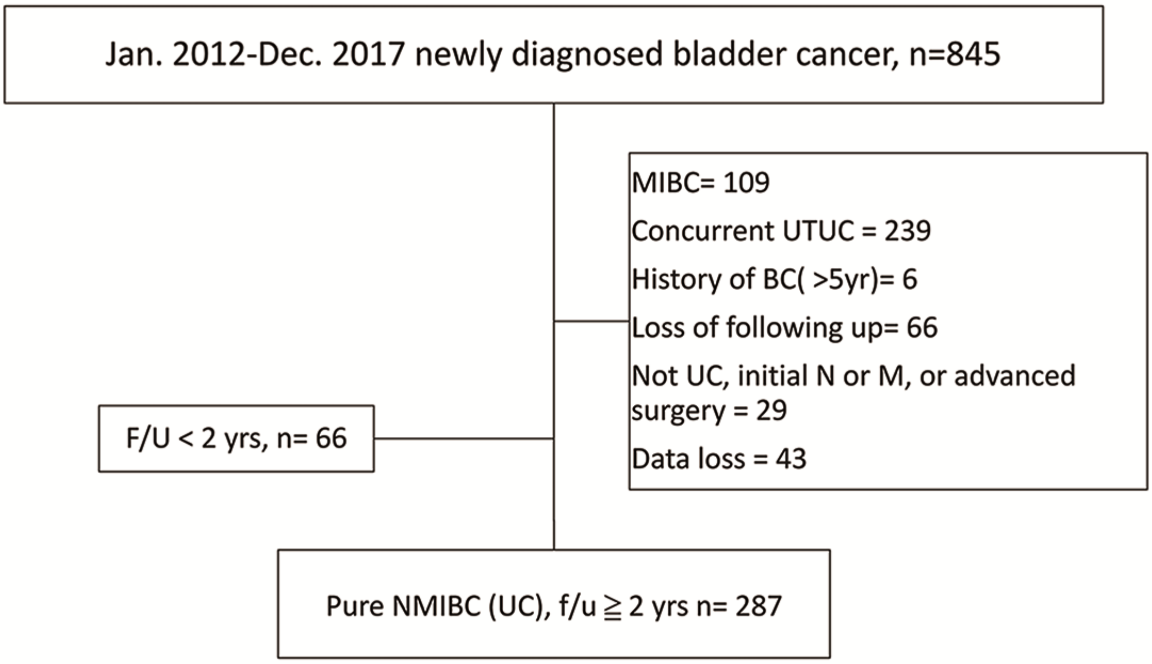

A total of 845 patients were screened during the study period. Of these patients, 353 patients were classified as “pure NMIBC“, with 287 patients who had a follow-up period of more than 2 years.

FIG. S2 Kaplan-Meier analysis of PFS

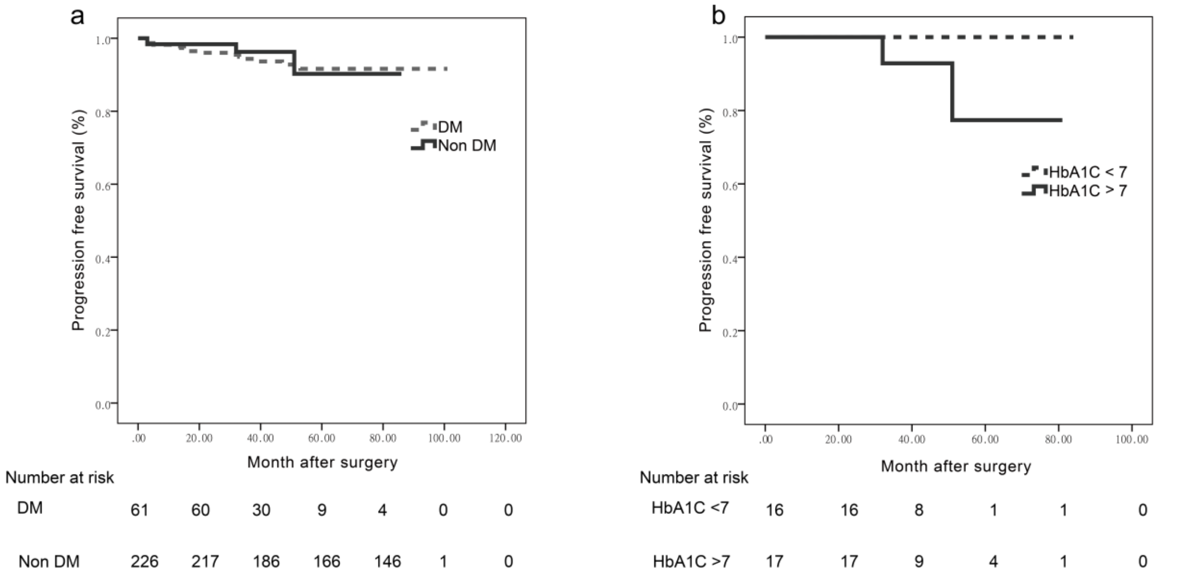

(a) PFS in the DM (median not reach) and non-DM groups (median not reach), log-rank *P*= 0.706.

(b) PFS between the proper glycemic control (median not reach) and the poor glycemic control groups (median not reach), log-rank *P*= 0.218.

Table S1 Characteristics of the DM and non-DM groups.

	No DM (n=226)		DM (n=61)		
	n	(%)	n	(%)	*P*
**Age, years (median)**	66		72		0.003*
**Sex**					
**Male**	170	(75.20%)	48	(78.70%)	0.574
**Female**	56	(24.80%)	13	(21.30%)	
**Smoking**	88	(41.30%)	22	(37.30%)	0.577
**BMI >24 kg/m^2**	114	(50.40%)	43	(71.70%)	0.003*
**Hypertension**	107	(47.30%)	52	(85.20%)	< 0.001*
**Cre >1.5 mg/dL**	25	(11.10%)	13	(21.30%)	0.036*
**Dialysis**	8	(3.50%)	2	(3.30%)	0.921
**History of other cancers**	23	(10.20%)	7	(11.50%)	0.769
**T stage**					
**cTa**	145	(64.20%)	34	(56.70%)	0.484
**cT1**	77	(34.10%)	24	(40.00%)	
**Pure CIS**	4	(1.80%)	2	(3.30%)	
**Concurrent CIS**	42	(18.60%)	16	(26.20%)	0.187
**High grade**	130	(58.30%)	42	(68.90%)	0.135
**Tumor number ≥3**	82	(46.90%)	20	(40.80%)	0.453
**Tumor size ≥3 cm**	35	(28.20%)	14	(42.40%)	0.118
**Intravesical therapy**	221	(97.80%)	58	(95.10%)	0.255
**BCG**	35	(15.50%)	12	(19.70%)	0.433
**Recurrence**	81	(35.80%)	28	(45.90%)	0.151
**Progression**	15	(6.60%)	3	(4.90%)	0.623

Characteristics were similar between the two groups in terms of sex, smoking rate, dialysis rate, clinicopathologic features of tumors, intravesical treatments, recurrence rate, and progression rate. Median age at the diagnosis of bladder cancer was older in the DM group. BMI, hypertension rate, and renal insufficiency rate were higher in the DM group.

BCG: Bacillus Calmette–Guérin; BMI: body mass index; CIS: carcinoma in situ; DM: diabetes mellitus; * $P < 0.05$

Table S2

Title: Characteristics in the proper glycemic control group and the poor glycemic control group.

	HbA1C ≤7 (n=16)		HbA1C ≥7 (n=17)		
	n	(%)	n	(%)	*P*
**Age, years (median)**	74		69		0.228
**Sex**					
**Male**	12	(75.00%)	16	(94.12%)	0.175
**Female**	4	(25.00%)	1	(5.88%)	
**Smoking**	6	(37.50%)	7	(43.75%)	0.719
**BMI >24 kg/m^2**	12	(75.00%)	13	(76.47%)	1.000
**Hypertension**	15	(93.75%)	14	(82.35%)	0.316
**Cre >1.5 mg/dL**	1	(6.25%)	7	(41.18%)	0.039*
**Dialysis**	0	(0.00%)	2	(11.76%)	0.485
**History of other cancers**	5	(31.25%)	1	(5.88%)	0.085
**OHA**					
**Metformin**	11	(68.75%)	11	(64.70%)	0.805
**TZD**	1	(6.25%)	1	(5.88%)	0.965
**T stage**					
**cTa**	9	(56.25%)	9	(52.94%)	0.616
**cT1**	7	(43.75%)	7	(41.18%)	
**Pure CIS**	0	(0.00%)	1	(5.88%)	
**Concurrent CIS**	7	(43.75%)	3	(17.65%)	0.141
**High grade**	13	(81.25%)	10	(58.82%)	0.259
**Tumor number ≥3**	6	(50.00%)	6	(42.86%)	1.000
**Tumor size ≥3 cm**	4	(44.44%)	4	(40.00%)	1.000
**Intravesical therapy**	14	(87.50%)	16	(94.12%)	0.509
**BCG**	2	(12.50%)	4	(23.53%)	0.656
**Recurrence**	4	(25.00%)	11	(64.71%)	0.037*
**Progression**	0	(0.00%)	2	(11.76%)	0.485

Renal insufficiency rate and recurrence rate were significantly higher in the poor glycemic control group.

BCG: Bacillus Calmette–Guérin; CIS: carcinoma in situ; OHA: oral hypoglycemic agent; TZD: thiazolidinedione; * $P < 0.05$
